# Chemically Functionalised Graphene FET Biosensor for the Label-free Sensing of Exosomes

**DOI:** 10.1038/s41598-019-50412-9

**Published:** 2019-09-26

**Authors:** Deana Kwong Hong Tsang, Tyler J. Lieberthal, Clare Watts, Iain E. Dunlop, Sami Ramadan, Armando E. del Rio Hernandez, Norbert Klein

**Affiliations:** 1 Department of Materials, Imperial College London, London, SW7 2AZ UK; 2 Department of Bioengineering, Imperial College London, London, SW7 2AZ UK

**Keywords:** Sensors and biosensors, Two-dimensional materials

## Abstract

A graphene field-effect transistor (gFET) was non-covalently functionalised with 1-pyrenebutyric acid N-hydroxysuccinimide ester and conjugated with anti-CD63 antibodies for the label-free detection of exosomes. Using a microfluidic channel, part of a graphene film was exposed to solution. The change in electrical properties of the exposed graphene created an additional minimum alongside the original Dirac point in the drain-source current (*I*_ds_) *-* back-gate voltage (*V*_*g*_) curve. When phosphate buffered saline (PBS) was present in the channel, the additional minimum was present at a *V*_g_ lower than the original Dirac point and shifted with time when exosomes were introduced into the channel. This shift of the minimum from the PBS reference point reached saturation after 30 minutes and was observed for multiple exosome concentrations. Upon conjugation with an isotype control, sensor response to the highest concentration of exosomes was negligible in comparison to that with anti-CD63 antibody, indicating that the functionalised gFET can specifically detect exosomes at least down to 0.1 μg/mL and is sensitive to concentration. Such a gFET biosensor has not been used before for exosome sensing and could be an effective tool for the liquid-biopsy detection of exosomes as biomarkers for early-stage identification of diseases such as cancer.

## Introduction

Graphene has been of great interest since its discovery in 2004 by A. K. Geim and K. Novoselov^1^. Features such as its tunability, high mobility^2,3^ and optical transparency have facilitated graphene’s recent rise, and its unique combination of properties allows it to be tailored for many applications such as biosensors, gas sensors, capacitors and solar cells^4–6^. For biosensors, graphene can be used to identify and capture biomarkers: biomolecules, typically present in serum, saliva and tissues, that can provide information on the state and stage of a disease^7,8^. For such purposes, graphene’s sensitivity for biomarker detection could lead to earlier diagnosis and therefore better prognosis^9^.

Graphene field-effect transistors (gFETs) have been extensively studied for biosensor applications using several different geometries and gating methods. Graphene’s band structure results in a linear energy dispersion, which gives rise to two cones that cross at the Dirac point. When a voltage, *V*, is applied to gate, *g*, of the gFET structure, this is accommodated by a shift in the Fermi level (*E*_F_) away from the Dirac point leading to conduction and the field-effect describes the induced charge carriers’ dependency on the sign of the voltage applied. A positive *V*_g_ results in conduction by electrons whilst a negative *V*_g_ causes conduction by holes as the Fermi level is shifted to the conduction and valence band, respectively. Graphene used for gFETs itself can be fabricated in many ways, and by using chemical vapour deposition (CVD) it is possible to obtain large-area, wafer-scalable material^10,11^ suitable for integration with microfluidics to produce high-sensitivity lab-on-chip devices. Such devices based on gFETs take advantage of graphene’s ambipolar effect whereby conduction is allowed through both electrons and holes, which can be further modulated by chemical dopants. When used in biosensor applications a gFET’s sensitivity to charge and tunability can be used as an indication of the presence and concentration of specifically bonded species such as proteins and cells^12,13^.

Recently, there has been extensive interest in blood-circulating biomarkers that may allow a “liquid biopsy” for diagnostics as well as investigation of disease etiology. Among circulating biomarker candidates such as proteins, circulating tumour cells, and nucleic acids, a new class of biomarkers called extracellular microvesicles are gaining prominence^14,15^. One subpopulation of extracellular vesicles is exosomes which are vesicles 40–150 nm in size expelled by both healthy and tumour cells^16^. Exosomes contain an abundance of genetic information about the cell from which they are derived and hence can be used as biomarkers for the early-stage detection of cancer^17,18^. Unlike other biomarkers such as circulating tumour cells, exosomes are present in blood serum in high numbers (10^6^–10^11^ per mL)^19,20^, however isolating them in high yield in order to interrogate vesicle protein abundance requires complex and time-consuming techniques. While conventional exosome analysis is conducted using flow cytometry, Western blot, or polymerase chain reaction, new techniques and devices have been described ranging from immunofluorescence, colorimetry, surface plasmon resonance (SPR), micro-nuclear magnetic resonance, and electrochemistry^21–25^. A majority of on-chip methods for either the isolation or detection of exosomes are not label-free, instead relying on conjugated beads or secondary antibodies for signal amplification, with the notable exception of SPR.

In this study, we fabricated a back-gated gFET biosensor using non-covalently functionalised CVD monolayer graphene. By chemically modifying graphene with antibodies, we tailored the graphene sensor surface for the specific label-free detection of exosomes. A microfluidic channel was used to expose only part of the graphene film to an analyte. The area of graphene exposed to the analyte and the area which remained unexposed exhibited different electrical properties, which resulted in an additional conductance minimum alongside the Dirac point when a current, *I*, was measured between the source, *s*, and drain, *d*, electrodes (*I*_ds_) while the voltage applied to the back gate (*V*_g_) was swept^26^. This has previously been reported to have been achieved by using both substitutional and adsorbate doping^27,28^. To the best of our knowledge, the double conductance feature has not yet been implemented for biosensing applications. The appearance of this feature and its change upon the introduction of exosomes to the graphene surface allows for their specific detection.

## Results and Discussion

**Figure 1:**
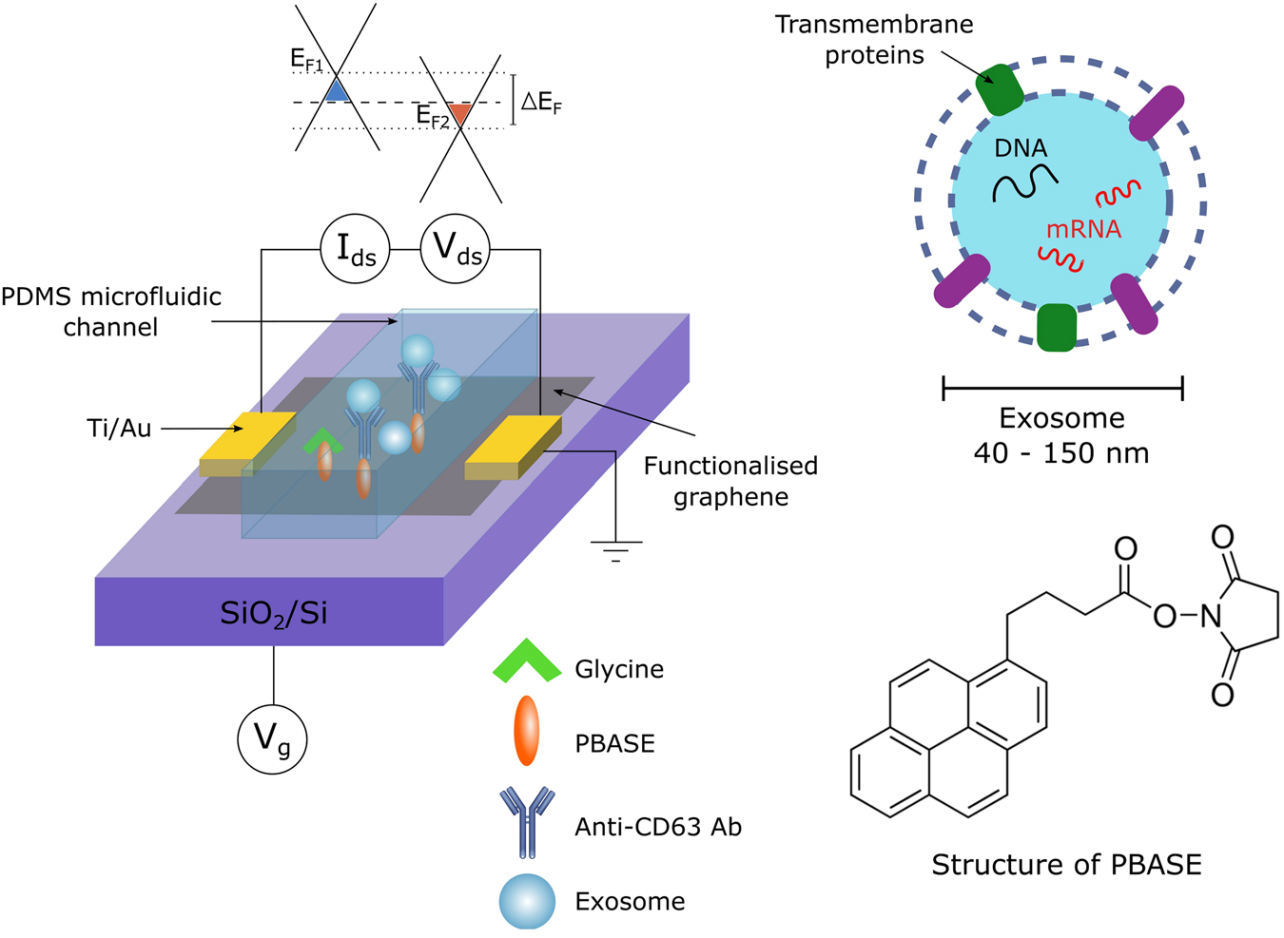
Schematic of the functionalised biosensor with microfluidic integration, showing the different doping levels of the covered and uncovered graphene as a result of exosome binding. Schematic of the basic structure of an exosome and molecular structure of 1-pyrenebutyric acid N-hydroxysuccinimide ester (PBASE).

Figure 1 shows a schematic of the sensor set-up, showing the microfluidic integration and required layers of functionalisation. The transfer of graphene to SiO_2_/Si substrates resulted in continuous, near fully monolayer graphene. This is shown in Supplementary Fig. S1, where only small islands of bilayer and multilayer graphene are observed, which may result in areas with different electrical properties. However, the advantage of large-area CVD graphene is that these areas are small in comparison to the overall size of the film and hence cause no major disruption to the bulk properties or overall device performance. Figure 1 also shows the molecular structure of 1-pyrenebutyric acid N-hydroxysuccinimide ester (PBASE), which is a heterobifunctional linker. The pyrene group stacks with graphene by π − π overlap to form a self-assembled monolayer with homogenous coverage^29^, whilst the N-hydroxysuccinimide (NHS) ester is able to react with primary amines present on a range of biomolecules, such as antibodies.

**Figure 2:**
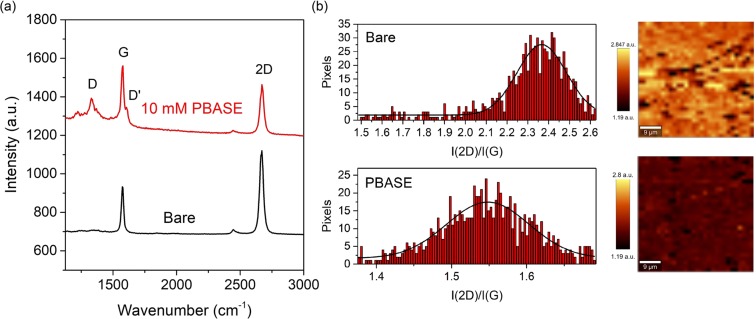
(**a**) Raman spectra of bare (black trace) and 10 mM PBASE functionalised (red trace) graphene with peak identifiers. The resulting spectra are averages of spectra taken from various points over the samples. (**b**) Comparison of the *I*(2D)/*I*(G) intensity ratio between bare and PBASE functionalised graphene where histograms were fitted with Gaussian curves and (**c**) the corresponding 2D/G Raman intensity ratio maps before and after functionalisation.

To confirm the presence of PBASE on the surface of the gFET sensor surface, both Raman spectroscopy and X-ray photoelectron spectroscopy (XPS) were employed. The resulting spectra in Fig. 2 for bare and PBASE-functionalised graphene were obtained by averaging multiple Raman spectra obtained at different positions on each sample, to give an overall view of the quality of the graphene after functionalisation with PBASE. Before functionalisation, the average intensity ratio of the 2D and G peak, *I*(2D)/*I*(G)^30,31^, is 2.37 and this decreases after functionalisation with PBASE to a value of 1.55 (Fig. 2(b)), indicating that there is some disorder present on the graphene surface. This is further supported by the presence of the D peak at 1350 cm^−1^, which is commonly attributed to the presence of defects in the graphene structure^31,32^, and the D’ peak that arises as a shoulder to the G peak (Fig. 2(a)), which is associated with surface charges and impurities. Spectra were taken and averaged over 4 samples before and after PBASE functionalisation. All 4 spectra for PBASE-functionalised samples are seen in Supplementary Fig. S2, where the resulting spectra exhibit high reproducibility due to similar peak intensity ratios and the presence of the D and D’ peaks.

**Figure 3:**
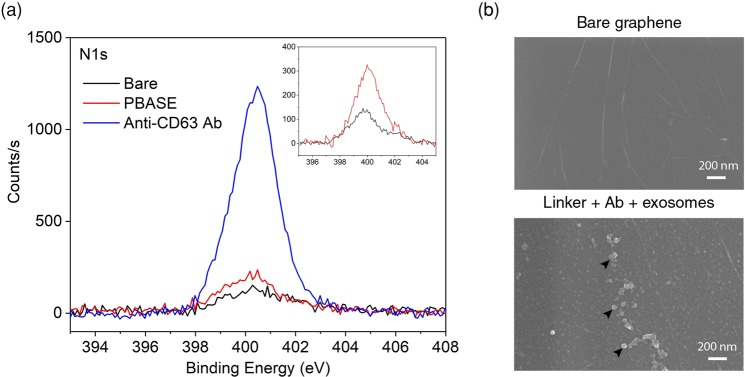
(**a**) N1s XPS spectrum of graphene with different levels of functionalisation: bare, PBASE and with PBASE + anti-CD63 Ab. Inset: N1s XPS spectrum of bare and PBASE functionalised graphene (**b**) SEM images of bare and anti-CD63 Ab conjugated graphene with adherent exosomes (arrowheads).

XPS analysis of functionalised samples can be used to confirm the presence of PBASE on the graphene surface by analysis of the N1s peak at 400.1 eV. This can be seen in Fig. 3(a) where the increase in intensity of the N1s peak suggests successful functionalisation^33^, since the only source of nitrogen atoms in functionalised graphene is the PBASE molecule. Still, the intensity of the N peak remains small compared to that of carbon due to the presence of only one N atom per PBASE molecule. The combination of Raman spectroscopy and XPS hence confirms that PBASE is present on the graphene surface. Supplementary Fig. S3 shows the resulting Raman spectrum from the functionalisation using a diazonium salt, which is reported to be a covalent linker for graphene-based biosensors^34^. 4-nitrophenyl diazonium (4 - NPD) was introduced to the graphene surface and Supplementary Fig. S3 shows the dramatic change in *I*(2D)/*I*(G) from 1.81 to 0.36 and significant presence of the D peak. The significant peak intensity of the D peak with respect to the G and 2D peak suggests a high density of defects introduced to the sample^35^, which could limit the overall performance of the device. For this reason, non-covalent functionalisation was chosen as most appropriate for the sensor. The Raman spectrum from PBASE-functionalised graphene does not show disorder to this extent and suggests non-covalent functionalisation at the surface typically by π-π stacking between the pyrene group and graphene’s hexagonal plane^7^.

The PBASE linker molecules subsequently bond to anti-CD63 antibodies (Ab), to make the sensor surface suitable for the specific capture of exosomes. The exosomal membrane protein CD63 is a tetraspanin commonly used in identification and isolation of exosomes^36^. After Ab conjugation, the sensors were further passivated with glycine to terminate unreacted NHS groups on the PBASE molecules to prevent the undesired, non-specific binding of exosomes directly to PBASE. Supplementary Fig. S4 shows the *I*_ds_ – *V*_ds_ and *I*_ds_ − *V*_g_ curves after each stage of functionalisation and suggests that the graphene still retains its expected electrical characteristics after each step. The presence of antibodies after conjugation was confirmed using XPS by the increase in N1s intensity with respect to PBASE only functionalised samples, as shown in Fig. 3(a), which can be attributed to the large number of amine and amide groups present on the antibodies^37,38^. Exosomes held on the graphene surface after being introduced to anti-CD63 Ab conjugated samples can be clearly observed by scanning electron microscopy (SEM) as seen in Fig. 3(b). From the image, the sizes of the exosomes present on the surface were found to be 94.1 ± 31.4 nm, which is consistent with values in literature^15,39^.

**Figure 4:**
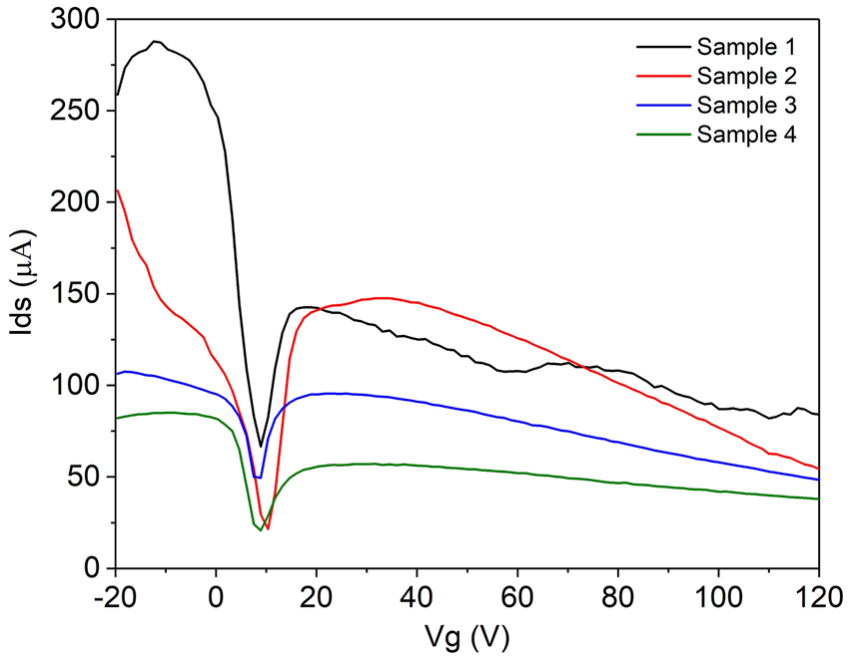
*I*_ds_ − *V*_g_ curve of 4 identically fabricated and functionalised gFET sensors with PBS present in the microfluidic channel.

The microfluidic channel was filled with phosphate buffered saline (PBS) so that only the graphene under the channel is exposed to the charged species in solution. As a result, this area exclusively becomes doped due to charge modulation^26,40,41^. This effect is observed as an additional conductance minimum in the *I*_ds_ − *V*_g_ curve at a different value of *V*_g_ than that of the bare graphene Dirac point. This arises due to the change in resistance of the graphene in the area under the microfluidic channel resulting in two regions of different resistance in the graphene film between source and drain. The combination of these two resistances manifests as the superposition of two *I*_ds_ − *V*_g_ curves, seen as two separate minima. The charged species in solution dope the graphene so that the uncovered graphene remains p-doped and the graphene exposed to the solution is selectively n-doped. Hence, a higher density of electrons is induced in the graphene film under the channel. The additional minimum for each sensor appears at similar values of *V*_g_ with average position of 9.3 ± 0.7 V, suggesting a near universal response of the sensor to PBS as seen in Fig. 4. The position of additional minimum that arises from graphene exposed to PBS is much lower than the Dirac point of the unexposed graphene, which suggests that PBS n-dopes the exposed area due to the screening of negative charges at the surface (that otherwise results in p-doping of the unexposed graphene).

Graphene’s electrical properties are modified upon the presence of chemical dopants and adsorbates. Hence, it is expected that when the graphene under the microfluidic channel is introduced different charged species, the position of the additional minimum would be affected. To determine sensor response, PBS and exosomes in PBS were introduced to the microfluidic channel. The relative position of the additional minimum, with respect to its position under PBS condition (*V*_PBS_) was then monitored rather than its absolute position. Changes in graphene quality, surface adsorbates and chemical dopants will vary from sample to sample, affecting the mobility and therefore the observed shape of the *I*_ds_ − *V*_g_ curve^42^, however the position of the Dirac point, as well as the additional minimum induced by the presence of PBS and exosomes is unaffected by such changes and therefore provides the basis of our sensing mechanism.

**Figure 5:**
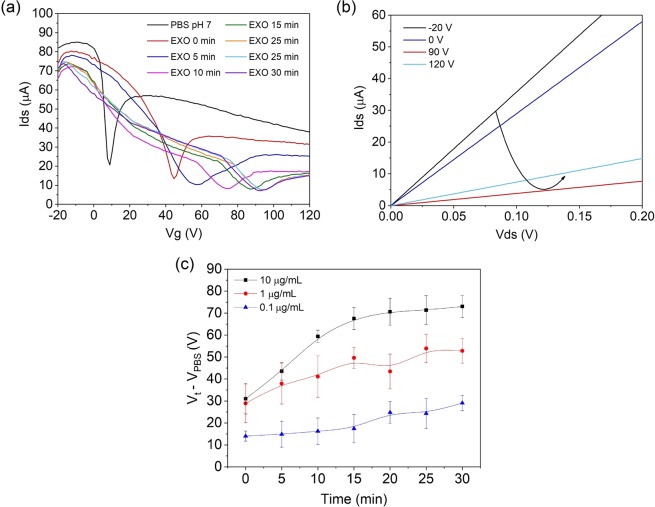
(**a**) *I*_ds_ − *V*_g_ curve of a functionalised gFET sensor with PBS (black trace) and 10 μg/mL exosomes in 0.001X PBS measured at room temperature over 30 minutes (coloured traces). (**b**) *I*_ds_ − *V*_ds_ measurement for the preliminary 10 μg/mL measurement at various constant *V*_g_ after 30 minutes of exosome binding. (**c**) Shift in the additional minimum relative to the PBS reference value for 3 different exosome concentrations performed over 3 samples each, where *V*_t_ − *V*_PBS_ values were averaged for each time point. Error bars denote ± one standard deviation of averaged *V*_t_ − *V*_PBS_ values obtained for 3 samples for each concentration.

The sensor response was investigated with an exosome concentration of 10 μg/mL in PBS pH 7, which was left if the channel for 30 minutes to ensure Ab binding, and *I*_ds_ − *V*_g_ curves recorded every 5 minutes. The position of the additional conductance minimum was as expected for PBS; however, a positive shift of this minimum was observed when exosome solutions were introduced to the channel, where *V*_t_ is the position of the additional minimum at each time point. Figure 5(a) shows a resulting *I*_ds_ − *V*_g_ curve in which there is a shift in the minimum over time where, after 30 minutes, the position (*V*_t=30_) has moved 83.1 V from *V*_PBS_ to 92.0 V. Similar responses were observed across 3 independent samples as shown in Supplementary Fig. S5. This positive shift correlates with the field-effect, whereby negatively charged exosomes induces positive charge in the graphene and cause p-doping respective to when only PBS is present. When part of the graphene is exposed to either PBS or exosomes, different levels of doping arise in the same graphene film. Despite this, by conducting an *I*_ds_ − *V*_ds_ measurement as seen in Fig. 5(b), the non-rectifying, linear behaviour typical of what is expected from monolayer graphene, is preserved^43^. The drop in *I*_ds_ at *V*_g_ = 90 V corresponds to the additional conductance minimum in Fig. 5(a).

As exosomes are first introduced into the microfluidic channel, they exist as a suspension of like-charged particles and therefore mutual repulsion ensures that they are homogeneously distributed in solution. As they bind to the anti-CD63 Ab conjugated surface, an inhomogeneous distribution of charges arises as exosomes are retained close to the graphene. This accumulation of charge causes the modulation of graphene’s electronic properties. This occurs over time due to the random movement of the exosomes in solution and eventually results in an established equilibrium over time. Figure 5(c) shows the relative shift in *V*_t_ from *V*_PBS_ (*V*_t_ – *V*_PBS_) for different exosome concentrations, which were obtained using the average of 3 samples measured under the same conditions. Here, a ‘saturation’ in the shift of *V*_t_ is seen for all exosome concentrations, which we associate with an equilibrium between exosome attachment and detachment after 30 minutes. Due to the excessive concentration of Ab used, each exosome is likely to be bound to multiple anti-CD63 antibodies, hence the detachment is assumed to be low and not considered a problem in microfluidics^44^.

Figure 5(c) also shows the sensitivity of the gFET to exosome concentrations down to 0.1 μg/mL, whereby after 30 minutes the maximum *V*_t_ – *V*_PBS_ shift is higher for higher exosome concentrations. The lowest concentration of exosomes detected corresponds approximately to 5000 exosomes/μL based on the number of particles in the stock solution as provided by the supplier. Additionally, due to the concentration and number of exosomes present at 10 μg/mL, the saturation cannot be due to the close packing of the exosomes on the sensor surface. This is confirmed by Fig. 5(c), which shows that the saturation in the shift of the additional minimum is consistent for each different exosome concentration, but the magnitude of the *V*_t_ – *V*_PBS_ shift at which this saturation occurs is dependent on exosome concentration. The response of the gFET sensor also correlates to realistic concentrations of exosomes in liquid biopsies^19,20^ and is comparable to current sensitivities of other exosome biosensors^45^.

**Figure 6:**
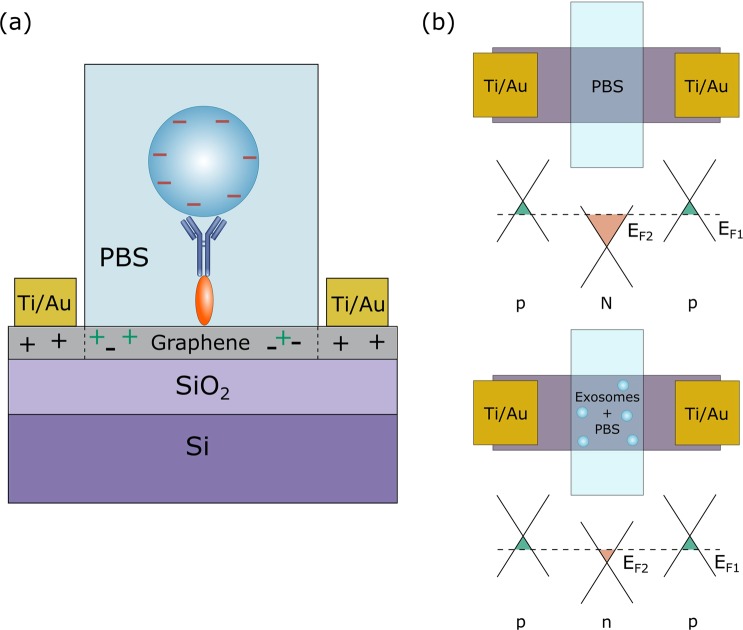
Schematic describing the gFET mechanism of sensing negatively charged exosomes when bound to the graphene surface. (**a**) The exosomes present in the microfluidic channel exhibit a negative surface charge. The PBASE linker and antibody constitute a physical separation between the exosome and graphene acting as a dielectric layer with thickness *d*. (**b**) Negative charge imparted by the exosomes causes positive charge accumulation in the graphene respective to the n-doping effect of PBS which results in a change in the Fermi energy.

For all exosome concentrations, the positive shift from the PBS position when exosomes are present, confirms the expected negative surface charge of exosomes at pH 7, as illustrated in Fig. 6(a) where *E*_F1_ and *E*_F2_ are the Fermi levels of the graphene not exposed to the solution and exposed to the solution, respectively (Fig. 6(b)). It is worth noting that in Fig. 6(a) the maximum thickness of the functionalisation layer, *d*, is estimated as 12 nm, given that the total molecular length of the PBASE linker is approximately 2 nm and the antibodies are 10 nm. The chosen PBS concentration of 0.001X PBS gives a Debye screening length of approximately 24 nm^46^, which is therefore large enough to contain the point of binding between the exosomes and antibodies.

**Figure 7:**
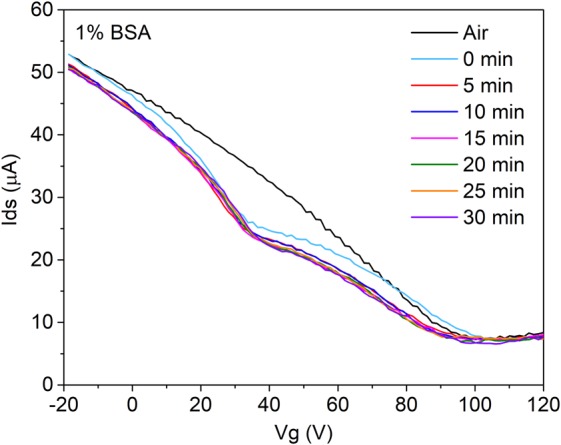
*I*_ds_ − *V*_g_ curve of a functionalised gFET sensor with air and 1% BSA in PBS measured over 30 minutes. For each time measurement, 3 measurements were taken, and their curves averaged.

To investigate the specificity of the sensor response to exosomes, a non-specific target protein, bovine serum albumin (BSA) in PBS, was introduced to the microfluidic channel. The typical Dirac point of the graphene sheet is observed at 105 ± 2.3 V (Fig. 7) when only air is present in the channel, which corresponds to the level of doping of the graphene after functionalisation with PBASE linkers and Ab conjugation. With BSA in PBS, the additional minimum appears at 37 ± 1.9 V, suggesting that when BSA in PBS is present in the channel the graphene underneath is overall more n-doped compared to that not exposed to the solution. As the isoelectric point of BSA is 5.4, at pH 7^47^, the proteins would be expected to carry negative charge. Given that PBS without BSA generally results in a minimum at approximately 10 V (Fig. 4), the positive shift of the additional minimum with BSA is consistent with the positive shift of the exosomes. The development of the additional conductance minimum and its shift from *V*_PBS_ shows that the graphene sensor is indeed responsive to exosomes and charged protein solutions; however, even though BSA was incubated in the channel over 30 minutes, Fig. 7 shows that the minimum does not appear to change in a time-dependent manner.

**Figure 8:**
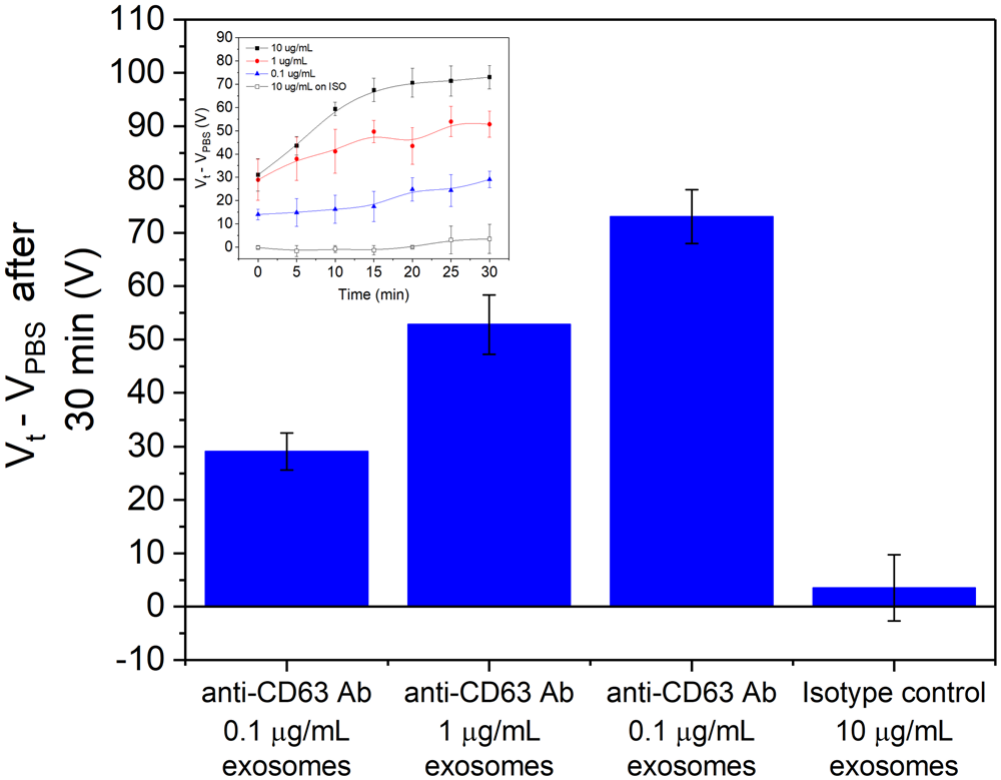
Shift in the additional minimum relative to the PBS reference after 30 minutes for anti-CD63 Ab-conjugated samples with exosomes of various concentrations alongside the shifts for the isotype-control conjugated samples with the maximum exosome concentration used (10 μg/mL exosomes). All 4 cases were performed over 3 samples with error bars denoting ± one standard deviation. Inset: *V*_t_ − *V*_PBS_ plot over time reproduced from Fig. 5(c) with additional curve for the isotype control with 10 μg/mL exosomes.

The lack of a time-dependent shift for BSA suggests that it does not specifically bind to the sensor surface. For exosomes, the saturation in the minimum’s position after 30 minutes is believed to be attributed to the reaching of an equilibrium in the binding of exosomes to the anti-CD63 Ab conjugated surface. To further investigate the specificity and confirm that the functionalisation performed corresponds to the specific binding of target exosomes, a non-specific antibody was conjugated to the surface instead of anti-CD63 Ab. The IgG1κ isotype control was conjugated after PBASE functionalisation and is identical to the anti-CD63 antibody except for the specific binding area, meaning that the target CD63 exosome membrane protein should not bind to it. Figure 8 shows the averaged response over 3 isotype sensors for 10 μg/mL exosomes, which can be directly compared to Fig. 5(c), which is the expected response to specific binding. From Fig. 8, there is negligible shift from *V*_PBS_ with exosomes on the isotype-conjugated sensor compared to the specific anti-CD63 Ab, which shows a significant *V*_t_ – *V*_PBS_ increase after 30 minutes at all exosome concentrations. The small change in *V*_t_ – *V*_PBS_ suggests that the isotype control does not bind to exosomes and gives a negligible signal, causing a small *V*_t_ – *V*_PBS_ maximum of 8.5 V after 30 minutes. This is observed across all 3 samples as seen from the *I*_ds_ − *V*_g_ curves in Supplementary Fig. S6. Comparing the saturation point after 30 minutes across all cases, as in Fig. 8, confirms that the anti-CD63 Ab conjugation results in a sensor that can specifically detect exosomes to concentrations of at least 0.1 μg/mL.

## Conclusion

In this study, back-gated gFETS were fabricated using monolayer graphene and chemically modified for the selective detection of exosomes. By using a PDMS microfluidic channel analytes can be introduced onto part of the graphene film. The area of graphene exposed to analyte and that unexposed are then observed to have different electrical properties. As a result, an additional minimum along with the original Dirac point of the graphene, appears on the *I*_ds_ − *V*_g_ curve. The position of this feature in relation to a reference point shifts over time to a saturation point after 30 minutes and is highly sensitive to low exosome concentrations of at least 0.1 μg/mL. By using BSA as a non-specific target, the anti-CD63 Ab conjugated sensor showed response to the presence of charged species but the additional minimum did not exhibit a time-dependent shift, which suggests that BSA does not bind to the sensor surface. The specificity of the sensor was further supported by isotype control experiments. These showed that the gFET sensor with isotype control antibodies did not exhibit a significant response compared to those conjugated with anti-CD63 Ab, suggesting that anti-CD63 Ab conjugation results in the specific detection of exosomes even at low concentrations. The lowest concentration that we detected is at the lower end of the range of the total number of exosomes expected in blood serum and comparable to other exosome sensors. Therefore, following optimisation, the gFET sensor could potentially be implemented for the specific detection of exosomes expelled from cancerous cell populations. Future work will involve the determination of the limit-of-detection and sensitivity of the gFET sensor, which could be investigated on microfabricated devices of well controlled graphene surface area. If low concentrations of exosomes expelled by cancerous cells can be specifically captured from a sub-millilitre liquid sample, the gFET sensor may prove to be a sensitive and useful tool for early-stage cancer diagnosis.

## Methods

### gFET device fabrication

Monolayer CVD graphene on 25 μm copper foil (GrollTex) was transferred onto a Si wafer with 300 nm thermal SiO_2_ (1–20 Ω cm and 525 μm thick) via a wet chemical transfer process whereby the copper foil was etched using 0.01 mg/mL ammonium peroxidisulphate ((NH_4_)_2_S_2_O_8_) solution. A sacrificial poly(methyl methacrylate) (PMMA) layer was used during this process, which was later washed off using dichloromethane. 10/50 nm Ti/Au electrodes were subsequently deposited by direct current (DC) magnetron sputtering for the gFET source and drain electrodes whereas the back-gate for the gFET was applied using conductive silver paint (RS electronics). A polydimethylsiloxane (PDMS) channel (10 mm length, 1 mm width and height and total volume of approximately 10 μL) was fabricated in-house and cured for 12 hours at room temperature (see Supplementary Fig. S7) before being secured on top of the gFET surface for microfluidic integration after sample functionalisation with a custom-made clamp.

### Functionalisation and antibody conjugation

After transfer onto Si wafers, the gFETs were incubated for 2 hours in PBASE in dimethylformamide (DMF) (Sigma-Aldrich) at room temperature before being rinsed in DMF and dried with N_2_. PBASE is a heterobifunctional linker that contains a pyrene group that stacks with graphene by π − π overlap and an N-hydroxysuccinimide (NHS) ester that reacts with primary amines. For the covalent functionalisation, 10 mM 4 – nitrophenyl diazonium salt (NPD) (Sigma Aldrich) with 0.1 M tetrabutylammonium tetrafluoroborate (n-Bu4NBF4) (Sigma Aldrich) was made up in acetonitrile. After transfer, samples were immersed in the absence of light for 15 hours with continuous stirring at room temperature. After functionalisation, the samples were then sonicated in acetonitrile for 1 minute to remove any excess diazonium salt. They were finally dried under a flow of N_2_.

Following PBASE functionalisation, samples were then conjugated with 100 μg/mL anti-CD63 antibody (BD Biosciences US). The antibodies are supplied in a stock solution of 0.5 mg/mL in an aqueous buffered solution (containing ≤0.09% sodium azide) and were diluted to 100 μg/mL using 1X PBS at pH 8.4. In order to conjugate these to PBASE NHS esters on the surface, gFET sensors were incubated by placing a 25 μL droplet of the antibody solution onto the surface and left overnight in a humidified environment at 4 °C. The sensors were then sequentially rinsed in 1X PBS at pH 8.4, de-ionised (DI) water and dried in air or under N_2_ flow. Similarly, for the isotype control conjugation, gFETs were incubated with purified mouse IgG1κ isotype control antibodies purchased from BD Biosciences US for specificity tests. These antibodies are not specific to exosomes but match the class and type of the anti-CD63 antibodies and hence act as negative controls. They are supplied at the same concentration and in the same aqueous buffer as anti-CD63 antibodies. All devices were subsequently rinsed with DI water before being treated with 100 mM glycine in 1X PBS pH 8.4 for 30 minutes for the termination of excess PBASE NHS groups at room temperature. After glycine treatment, samples were rinsed with DI water and dried with N_2_.

### Exosome reconstitution

The exosomes used in this study were purified lyophilized standards collected from healthy plasma donors using ultracentrifugation and microfiltration (Hansa Biomedical). Exosome solutions were reconstituted by adding 30 μL 0.001X PBS to the 30 μg lyophilized sample followed by gentle pipetting up and down 10–15 times for resuspension. The solutions were then vortexed for 60 seconds and centrifuged for 5 seconds at 3000 RPM. A final stock solution of 1 μg/μL was obtained from which other solutions were made by dilution with 0.001X PBS and stored at 4 °C. Exosome standards are reported to express CD63 both before lyophilization and after reconstitution of lyophilized samples.

### Electrical measurements

DC measurements were carried out using a two-channel Keithley 2636B sourcemeter. For each gFET sensor the *V*_g_ was swept between −20 and 120 V and supplied through the back-gate with a constant 200 mV source-drain voltage (*V*_ds_). The source-drain current (*I*_ds_) was measured for each *V*_g_ point and the gate current (*I*_gs_) was monitored to ensure negligible leakage current. The gFET responses were recorded when approximately 10 μL solutions of 0.001X phosphate buffered saline (PBS), bovine serum albumin (BSA) or exosomes in 0.001X PBS were perfused through a microfluidic channel. BSA was purchased as a lyophilized powder from Sigma Aldrich and made up to 1% solution in PBS and stored at 4 °C. 0.001X PBS was chosen for BSA and exosome solutions to minimize Debye screening. All DC measurements were performed at room temperature. For each exosome concentration studied, 3 samples were prepared and measured and the results averaged.

### Graphene characterisation

Raman spectroscopy was performed using a WITec Alpha300 RA spectrometer with a 532 nm laser operated at power <1 mW to avoid sample heating. To characterise the chemical composition of bare and functionalised graphene, X-ray photoelectron spectroscopy (XPS) analysis was carried out at room temperature using a high-throughput Thermo Fisher K-Alpha^+^ spectrometer with 400 μm spot size. For the scanning electron microscopy (SEM) of devices, exosomes were deposited on samples dropwise, fixed in 2.5% glutaraldehyde (Sigma Aldrich), lipid contrast stained with 1% OsO_4_ (Sigma Aldrich) for 1 hour, coated with 10 nm chromium, and imaged (Zeiss Sigma 300) at an operating voltage of 5 kV.

## Supplementary information

Supplementary Information

## References

[1] Novoselov KS (2004). Electric field effect in atomically thin carbon films. Science.

[2] Sarma SD, Adam S, Hwang EH, Rossi E (2010). Electronic transport in two dimensional graphene. Rev. Mod. Phys..

[3] Xia JL, Chen F, Wiktor P, Ferry DK, Tao NJ (2010). Effect of top dielectric medium on gate capacitance of graphene field effect transistors: implications in mobility measurements and sensor applications. Nano Lett..

[4] Yang M, Javadi A, Li H, Gong S (2010). Ultrasensitive immunosensor for the detection of cancer biomarker based on graphene sheet. Biosens. Bioelectron..

[5] Rumyantsev S, Liu G, Shur MS, Potyrailo RA, Balandin AA (2012). Selective gas sensing with a single pristine graphene transistor. Nano Lett..

[6] An Y, Behnam A, Pop E, Ural A (2013). Metal-semiconductor-metal photodetectors based on graphene/p-type silicon Schottky junctions. Appl. Phys. Lett..

[7] Zhou L (2017). Label-free graphene biosensor targeting cancer molecules based on non-covalent modification. Biosens. Bioelectron..

[8] Kim DJ (2013). Reduced graphene oxide field-effect transistor for label-free femtomolar protein detection. Biosens. Bioelectron..

[9] Myung S (2011). Graphene-encapsulated nanoparticle based biosensor for the selective detection of cancer biomarkers. Adv. Mater..

[10] Mattevi C, Kim H, Chhowalla M (2011). A review of chemical vapour deposition of graphene on copper. J. Mater. Chem..

[11] Li X (2009). Large-area synthesis of high-quality and uniform graphene films on copper foils. Science.

[12] Wang C (2016). A label-free and portable graphene FET aptasensor for children blood lead detection. Sci. Rep..

[13] Mao S (2013). Direct growth of vertically-oriented graphene for field-effect transistor biosensor. Sci. Rep..

[14] Chronopoulos A, Lieberthal TJ, del Río Hernández AE (2017). Exosomes as a platform for ‘liquid biopsy’ in pancreatic cancer. Converg. Sci. Phys. Oncol..

[15] Ko J, Carpenter E, Issadore D (2016). Detection and isolation of circulating exosomes and microvesicles for cancer monitoring and diagnostics using micro-/nano-based devices. Analyst.

[16] Kalluri R (2016). The biology and function of exosomes in cancer. J. Clin. Invest..

[17] Lee K, Shao H, Weissleder R, Lee H, Al LEEET (2015). Acoustic purification of extracellular microvesicles. ACS Nano.

[18] Melo SA (2015). Glypican-1 identifies cancer exosomes and detects early pancreatic cancer. Nature.

[19] Vlassov AV, Magdaleno S, Setterquist R, Conrad R (2012). Exosomes: Current knowledge of their composition, biological functions, and diagnostic and therapeutic potentials. Biochim. Biophys. Acta - Gen. Subj..

[20] Grasso L (2015). Molecular screening of cancer-derived exosomes by surface plasmon resonance spectroscopy. Anal. Bioanal. Chem..

[21] Kanwar SS, Dunlay CJ, Simeone DM, Nagrath S (2014). Microfluidic device (ExoChip) for on-chip isolation, quantification and characterization of circulating exosomes. Lab Chip.

[22] Vaidyanathan R (2014). Detecting exosomes specifically: a multiplexed device based on alternating current electrohydrodynamic induced nanoshearing. Anal. Chem..

[23] Im H (2014). Label-free detection and molecular profiling of exosomes with a nano-plasmonic sensor. Nat. Biotechnol..

[24] Jeong S (2016). Integrated magneto–electrochemical sensor for exosome analysis. ACS Nano.

[25] Shao H (2012). Protein typing of circulating microvesicles allows real-time monitoring of glioblastoma therapy. Nat. Med..

[26] Feng T (2014). Back-gate graphene field-effect transistors with double conductance minima. Carbon N. Y..

[27] Mattiuzzi A (2012). Electrografting of calix[4]arenediazonium salts to form versatile robust platforms for spatially controlled surface functionalization. Nat. Commun..

[28] Iqbal MZ, Anwar N, Siddique S, Iqbal MW, Hussain T (2017). Formation of pn-junction with stable n-doping in graphene field effect transistors using e-beam irradiation. Opt. Mater. (Amst)..

[29] Zhen XV (2018). Noncovalent monolayer modification of graphene using pyrene and cyclodextrin receptors for chemical sensing. ACS Appl. Nano Mater..

[30] Huang Y (2010). Nanoelectronic biosensors based on CVD grown graphene. Nanoscale.

[31] Wang Y, Chen X, Zhong Y, Zhu F, Loh KP (2009). Large area, continuous, few-layered graphene as anodes in organic photovoltaic devices. Appl. Phys. Lett..

[32] Kodali VK (2011). Nonperturbative chemical modification of graphene for protein micropatterning. Langmuir.

[33] Chang H-N, Sarkar S, Baker JR, Norris TB (2016). Fluorophore and protein conjugated Diels-Alder functionalized CVD graphene layers. Opt. Mater. Express.

[34] Tehrani Z (2014). Generic epitaxial graphene biosensors for ultrasensitive detection of cancer risk biomarker. 2D Mater..

[35] Dresselhaus MS, Jorio A, Souza Filho AG, Saito R (2010). Defect characterization in graphene and carbon nanotubes using Raman spectroscopy. Philos. Trans. A. Math. Phys. Eng. Sci..

[36] Théry C (2006). Isolation and characterization of exosomes from cell culture supernatants and biological fluids. Curr. Protoc. Cell Biol..

[37] Hosseinidoust Z, Van De Ven TGM, Tufenkji N (2011). Bacterial capture efficiency and antimicrobial activity of phage-functionalized model surfaces. Langmuir.

[38] Wang J (2006). Covalent immobilization of glucose oxidase on conducting ultrananocrystalline diamond thin films. Diam. Relat. Mater..

[39] Akagi T, Kato K, Hanamura N, Kobayashi M, Ichiki T (2014). Evaluation of desialylation effect on zeta potential of extracellular vesicles secreted from human prostate cancer cells by on-chip microcapillary electrophoresis. Jpn. J. Appl. Phys..

[40] Baltazar J (2012). Facile formation of graphene P-N junctions using self-assembled monolayers. J. Phys. Chem. C.

[41] Sun Y (2017). Poly (ethylene imine)-modulated transport behaviors of graphene field effect transistors with double Dirac points. J. Appl. Phys..

[42] Iddo Heller (2007). Identifying the Mechanism of Biosensing with Carbon Nanotube Transistors. Nano Lett..

[43] Guo SR (2011). Label free DNA detection using large area graphene based field effect transistor biosensors. J. Nanosci. Nanotechnol..

[44] Contreras-Naranjo JC, Wu H-J, Ugaz VM (2017). Microfluidics for exosome isolation and analysis: enabling liquid biopsy for personalized medicine. Lab Chip.

[45] He M, Zeng Y (2016). Microfluidic exosome analysis toward liquid biopsy for cancer. J. Lab. Autom..

[46] Sultan SM, de Planque MRR, Ashburn P, Chong HMH (2017). Effect of phosphate buffered saline solutions on top-down fabricated ZnO nanowire field effect transistor. J. Nanomater..

[47] Shi Q, Zhou Y, Sun Y (2008). Influence of pH and ionic strength on the steric mass-action model parameters around the isoelectric point of protein. Biotechnol. Prog..

