# Supplementary material for: Chemically Functionalised Graphene FET Biosensor for the Label-free Sensing of Exosomes
Source: Sci Rep. 2019 Sep 26;9:13946. doi: 10.1038/s41598-019-50412-9 (PMC6763426; doi:10.1038/s41598-019-50412-9)
Supplement: Supplementary file 1 — Supplementary Information [file 41598_2019_50412_MOESM1_ESM.pdf]

Supplementary Information

Chemically Functionalised Graphene FET Biosensor for the Label-free Sensing of Exosomes

Deana Kwong Hong Tsang[1,*], Tyler J. Lieberthal[2], Clare Watts[1], Iain E. Dunlop[1], Sami Ramadan[1], Armando E. Del Rio Hernandez[2], Norbert Klein[1]

[1]*Department of Materials, Imperial College London, London, SW7 2AZ, UK*

[1]*Department of Bioengineering, Imperial College London, London, SW7 2AZ, UK*

[*] dk2211@imperial.ac.uk

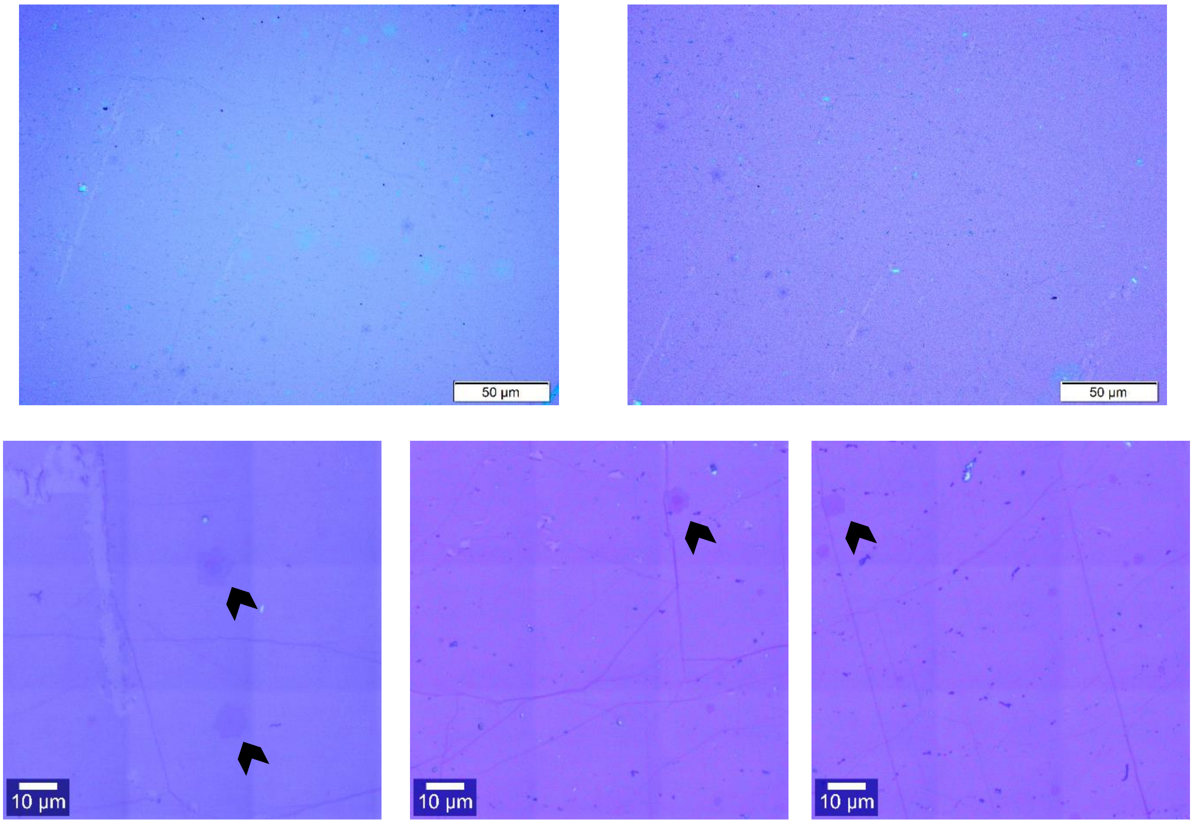

Supplementary figure S1: Optical micrographs of different graphene films from the same batch of CVD graphene transferred onto SiO_2/Si substrates. Some areas of bilayer or multilayer graphene are identified by the well-faceted shape and colour contrast and are indicated with arrowheads.

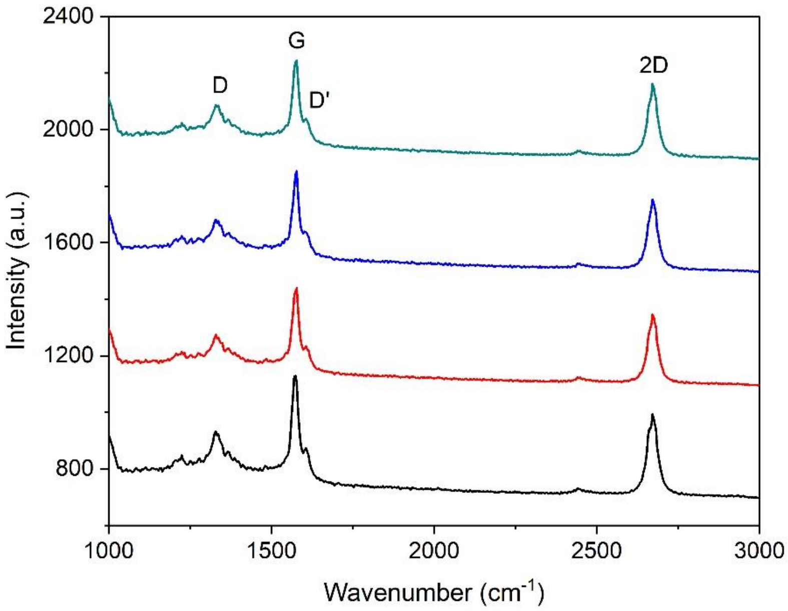

Supplementary figure S2: Raman spectra of 4 CVD graphene samples transferred onto SiO_2/Si substrates and functionalised with 10 mM PBASE. The resulting spectra are averaged spectra over measurements taken at multiple points for each sample.

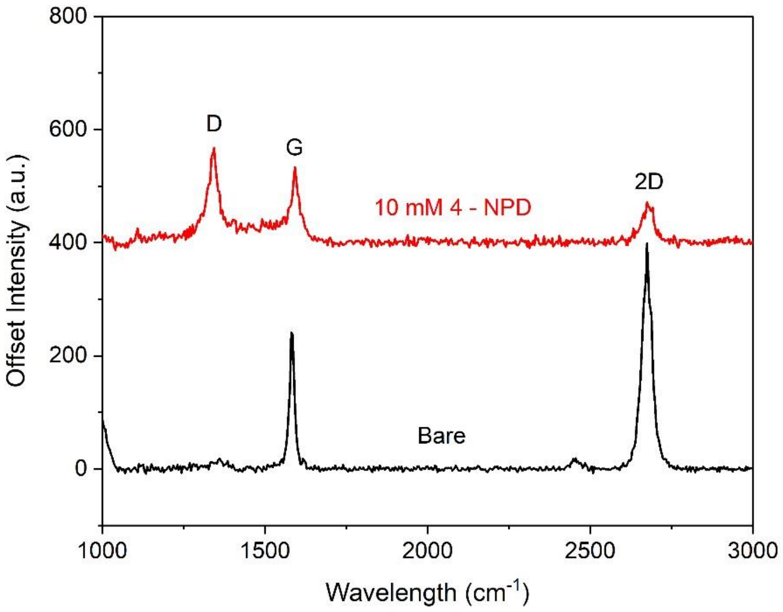

Supplementary figure S3: Raman spectra of a bare and 10 mM 4 - nitrophenyl diazonium salt functionalised CVD graphene sample transferred onto a SiO_2/Si substrate averaged over 4 points on the same sample.

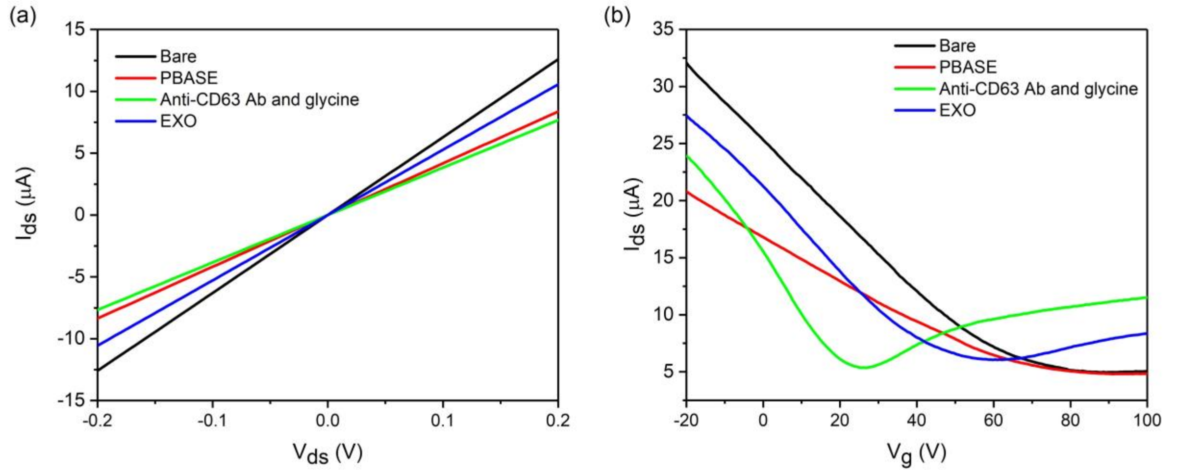

Supplementary figure S4: (a) $I_{ds} – V_{ds}$ and (b) I_{ds} - V_g curves for a gFET sensor after each stage of functionalisation and after introduction of 10^{-2} μg/μL exosomes in 0.001X PBS to the surface through a microfluidic channel.

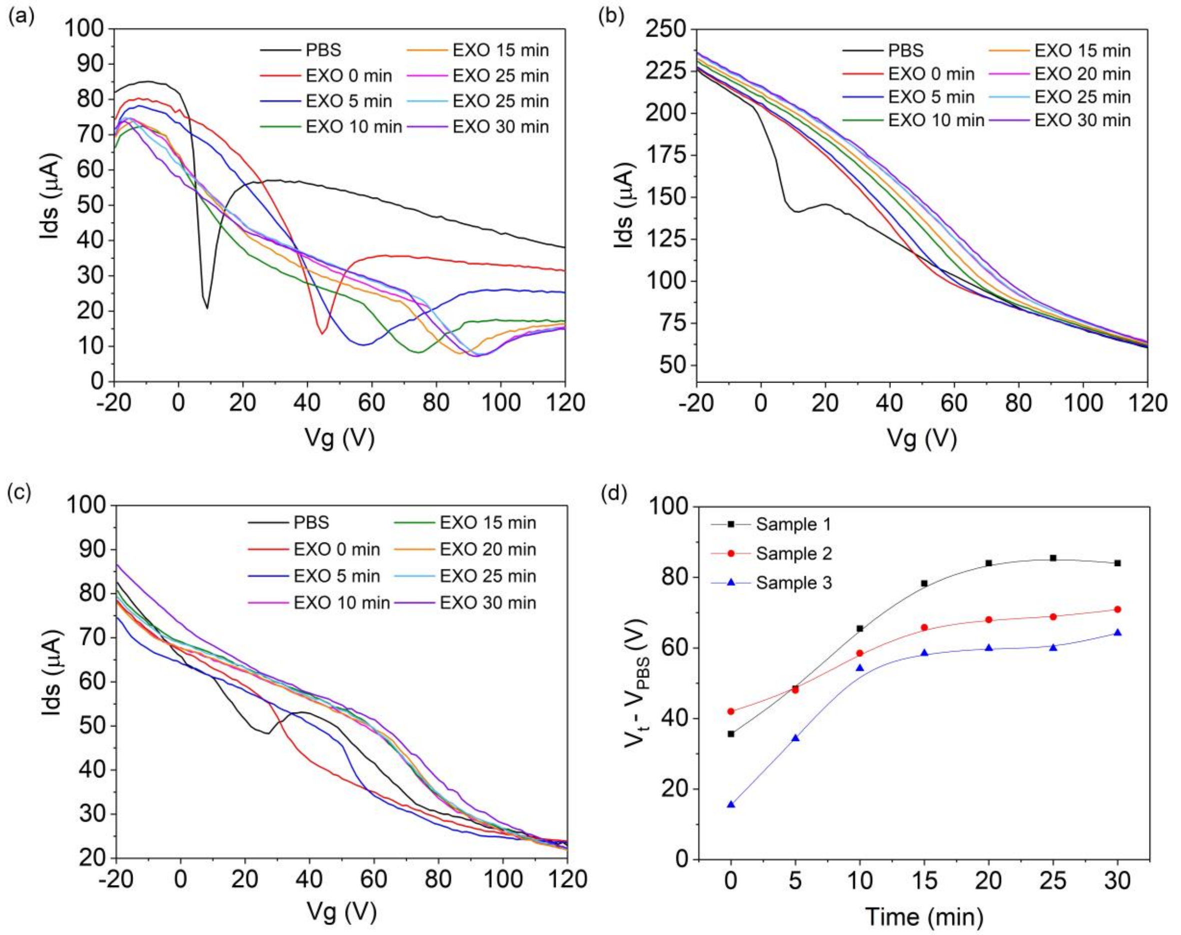

Supplementary figure S5: I_{ds} - V_g curves for 3 identically fabricated and functionalised gFET sensors and their response to 0.001X PBS and 10^{-2} μg/μL exosomes in 0.001X PBS pH 7. (a) Sample 1 as shown in the main manuscript, (b) sample 2 and (c) sample 3. (d) $V_t – V_{PBS}$ plot over time of all three sensors for a concentration of 10^{-2} μg/μL exosomes, which correspond to the relevant I_{ds} - V_g curves of sample 1, 2 and 3.

The curves in supplementary figure S5 show there is variation in the quality of the graphene and functionalisation between samples, however the relative shift of V_t with time is still similar between samples despite these differences. $V_t - V_{PBS}$ for each of these samples is still representative for the highest concentration of exosomes.

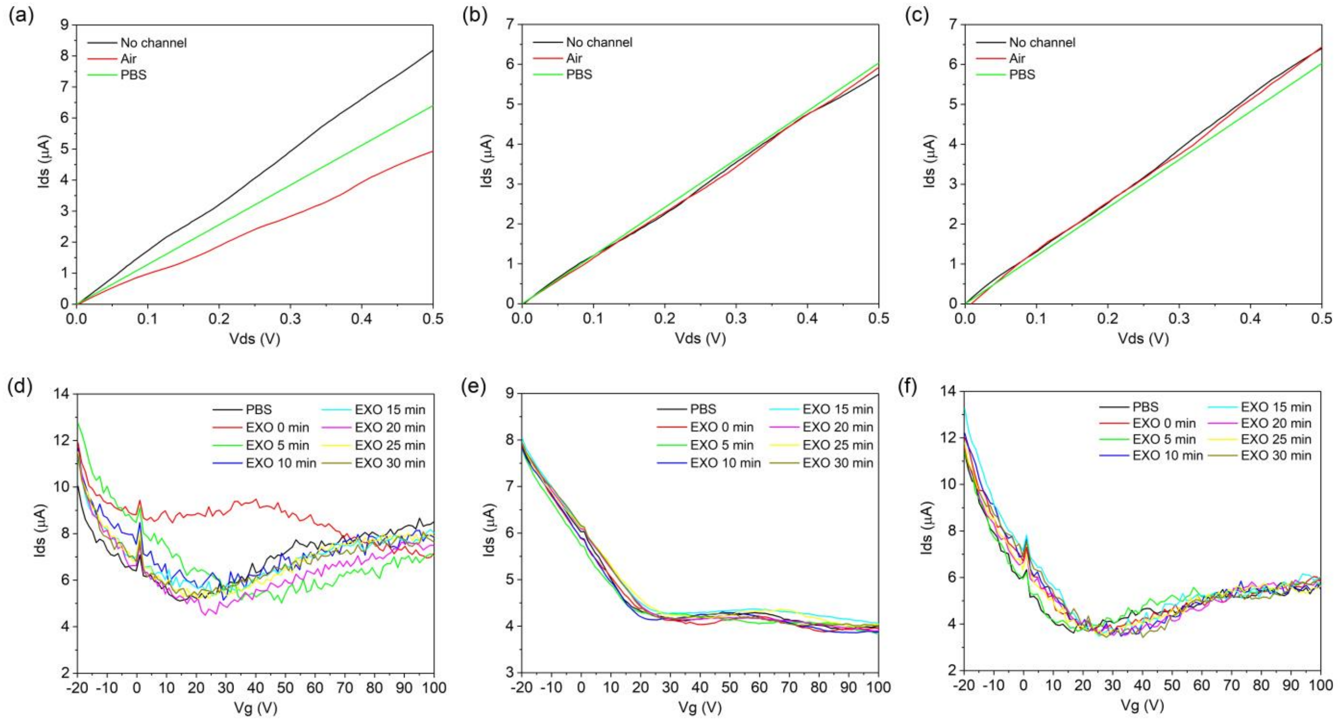

Supplementary figure S6: Isotype control experiments. $I_{ds} - V_{ds}$ measurement at V_g = 0 V of sample (a) 1, (b) 2 and (c) 3 and $I_{ds} - V_g$ curves for sample (d) 1, (e) 2 and (f) 3 over 30 minutes with introduction of 10^{-2} μg/μL exosomes in 0.001X PBS pH 7. All 3 gFET sensors were identically fabricated and functionalised with the isotype control antibody.

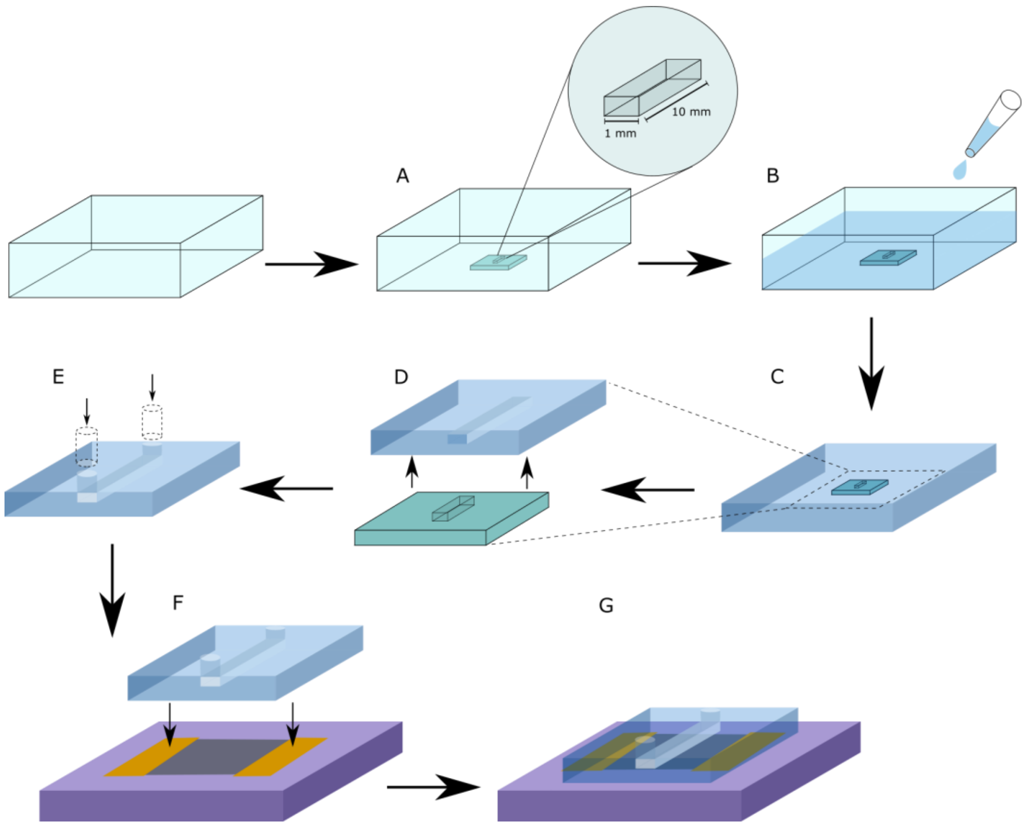

Supplementary figure S7: Schematic showing the step-by-step fabrication of polydimethylsiloxane (PDMS) microfluidic channels. A negative mould is 3D printed with the desired channel size and placed in a clean container (A). RTVA and RTVB are mixed together with the desired ratio and poured into the container, which is then left to cure overnight (B). The cured PDMS is then removed from the container and trimmed to a size appropriate for the gFET sensor such that excess PDMS is removed (C). The negative mould is then detached from the cured PDMS, which is left with the inverse shape of the negative mould (D). Inlet and outlets of the channel are created using a biopsy punch (E). The PDMS microlfuidic channel is finally cleaned and aligned (F) and placed on the surface of the gFET sensor (G).

The microfluidic channels were made using RTV 615 A + B polydimethylsiloxane (PDMS), which is supplied by Circuit Specialists Europe Ltd. as a two-component product mixed with a specific ratio to give the desired cured properties. The supplied PDMS is mostly made up by long chain vinyl-containing polydimethylsiloxane, vinyl-containing silicone resin, methyl hydrogen-siloxane, small amounts of solvent (benzene/toluene) and chloroplatinic acid which acts as a catalyst. In order to cure the PDMS, the two parts, A and B, were mixed with a recommended ratio of 10:1, where A is the vinyl-containing resin and B is the hydrosiloxane copolymer curing agent. For all channels used for measurements, this ratio was slightly altered to 12:1, reducing the curing agent, which resulted in a softer, more elastic PDMS that gave better adhesion to the gFET surface.
